# Control strategy of the novel stator free speed regulating wind turbine generation system

**DOI:** 10.1371/journal.pone.0314226

**Published:** 2024-12-06

**Authors:** Yanan Li, Jianhui Cui, Hongna Li, Bin Zhao

**Affiliations:** 1 Maritime College, Tianjin University of Technology, Tianjin, China; 2 Tianjin Mingyang Wind Power Equipment Co., Ltd. Tianjin, China; SRM Institute of Science and Technology (Deemed to be University), INDIA

## Abstract

Building a high-proportion renewable energy power system is a key measure to address the challenges of the energy revolution and climate change. However, current high-proportion renewable energy systems face issues of frequency instability and voltage fluctuations. To address these challenges, this paper proposes a novel topology for a stator free speed regulating wind turbine generation system. The stator free speed regulating machine connects a gearbox and a synchronous generator, forming a flexible drive chain that increases system inertia and enhances frequency stability in high-proportion renewable energy power systems. The synchronous generator at the end of the drive chain can be directly connected to the grid without the converter, thanks to the speed regulation provided by the stator free speed regulating machine, thus avoiding harmonic pollution caused by power electronic devices in traditional wind turbines, enhancing the reactive power support capability, and improving voltage stability in high-proportion renewable energy systems. Given that the proposed stator free speed regulating machine consists solely of a rotating inner and outer rotor without a stator, traditional motor control strategies are not applicable. Therefore, this paper focuses on developing control strategies for the stator free speed regulating machine, employing a dual closed-loop PI control strategy with an outer loop for speed and an inner loop for current, based on flux orientation of the outer rotor. Simulation experiments will validate the feasibility of the proposed stator free speed regulating wind turbine generation system topology and the effectiveness of the control strategy.

## 1. Introduction

To address global climate change, reduce greenhouse gas emissions, achieve sustainable energy development, and transform the energy structure, constructing a high-proportion renewable energy power system is an inevitable choice. During the process of building such a system, the power industry faces unprecedented challenges, among which frequency stability and voltage stability are the most prominent issues. These two indicators are fundamental to assessing the healthy operation of the power system, directly affecting the reliability of the power system and the quality of electrical energy supplied to users.

Frequency stability reflects the dynamic balance between electricity generation and consumption within the system. In traditional power systems, the rotational mechanical inertia of synchronous generators, such as those powered by thermal and hydroelectric energy, provides the necessary foundation for frequency stability [1]. This inertia effectively buffers the frequency fluctuations caused by sudden changes in load or generation, ensuring that the power system can quickly stabilize following disturbances [2]. However, renewable energy power systems, exemplified by wind energy, inherently exhibit intermittency and volatility, and the widespread use of power electronic devices significantly reduces the overall inertia of the power system [3]. This reduction manifests in two main ways: first, wind power generation equipment lacks the mechanical inertia of synchronous generators, which decreases frequency response time and results in rapid frequency fluctuations during supply-demand imbalances [4]; second, while the use of power electronic devices improves energy conversion efficiency, they do not provide inertia, further diminishing the system’s ability to withstand load fluctuations [5]. The absence of inertia leads to extended frequency recovery times and increased frequency deviations when the power system faces sudden load changes or faults, posing serious challenges to stability. Particularly during significant disturbances, this can result in frequency collapse, potentially triggering widespread blackout events [6].

Voltage stability is another critical indicator of the operational quality of the power system, directly impacting the quality of electrical energy supplied to users and the normal operation of electrical equipment. The issue of voltage instability in renewable energy power systems primarily stems from inadequate reactive power support [7]. Synchronous generators not only provide active power but can also adjust reactive power as needed to maintain voltage stability [8]. However, renewable energy devices, such as wind turbines, have limitations in providing reactive power. Wind turbines connect to the grid via power electronic converters, which limit their capacity to deliver reactive power, especially during voltage fluctuations [9]. Under full load, converters often cannot simultaneously meet maximum active and reactive power demands [10]. Additionally, the internal control strategies of wind turbines prioritize active power output, further constraining their reactive support capability [11]. During peak load conditions or system recovery from faults, the limited reactive power capability of wind turbines may be insufficient to meet voltage support requirements, leading to voltage sag [12]. Voltage fluctuations directly affect the operational stability of wind turbines; if the voltage drops, the turbines must absorb reactive power, exacerbating the voltage decline and creating a vicious cycle [13]. Furthermore, during faults or significant disturbances, inadequate reactive response from turbines may result in slow voltage recovery, potentially leading to voltage collapse [14].

Therefore, to ensure the stability and reliability of high-proportion renewable energy power systems, enhancing the system inertia and reactive support capabilities of wind power generation is imperative. Given the structural limitations of existing wind energy systems, pursuing breakthroughs through traditional technological avenues may prove challenging. To this end, researchers have proposed the concept of speed regulating wind turbines, which alter the structure of wind turbines by using speed regulating devices to connect the gearbox and synchronous generator [15]. This allows variable rotational speeds to be adjusted to a constant speed, achieving a flexible connection within the drive chain, thereby improving the system’s resistance to impact loads and increasing inertia to enhance frequency stability [16]. The synchronous generator at the end of the drive chain can connect directly to the grid without a converter, avoiding harmonic pollution associated with power electronic devices, thereby enhancing the system’s reactive support capacity to improve voltage stability [17]. Existing speed regulating wind turbines primarily fall into four categories: those based on differential gearing, hydraulic transmission, continuously variable transmission, and electromagnetic coupling.

### (1) Differential gear-based speed regulating wind turbine

**Fig 1:**
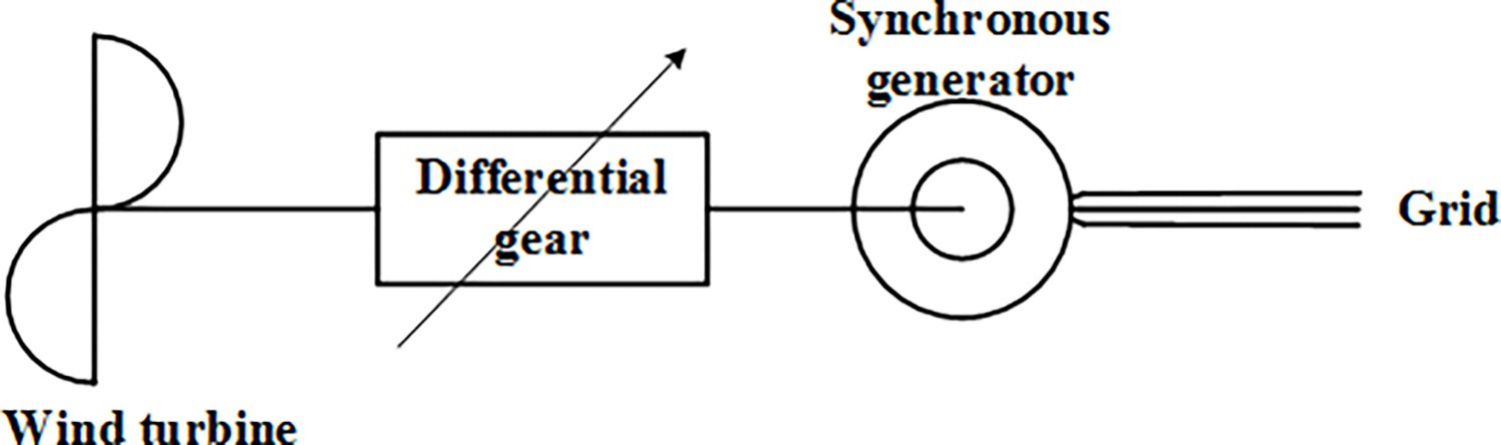
Structural diagram of the differential gear-based speed regulating wind turbine.

The structural schematic of the differential gear-based speed regulating wind turbine is shown in Fig 1 [18–21].

The differential gear-based speed regulating wind turbine achieves a flexible connection in the transmission chain through mechanical coupling, which increases system inertia and alleviates the impact of shock loads on the turbine to some extent. However, the high requirements for gear precision, material quality, bending strength, and temperature adaptability lead to increased material costs and processing difficulties.

### (2) Hydraulic drive speed regulating wind turbine

**Fig 2:**
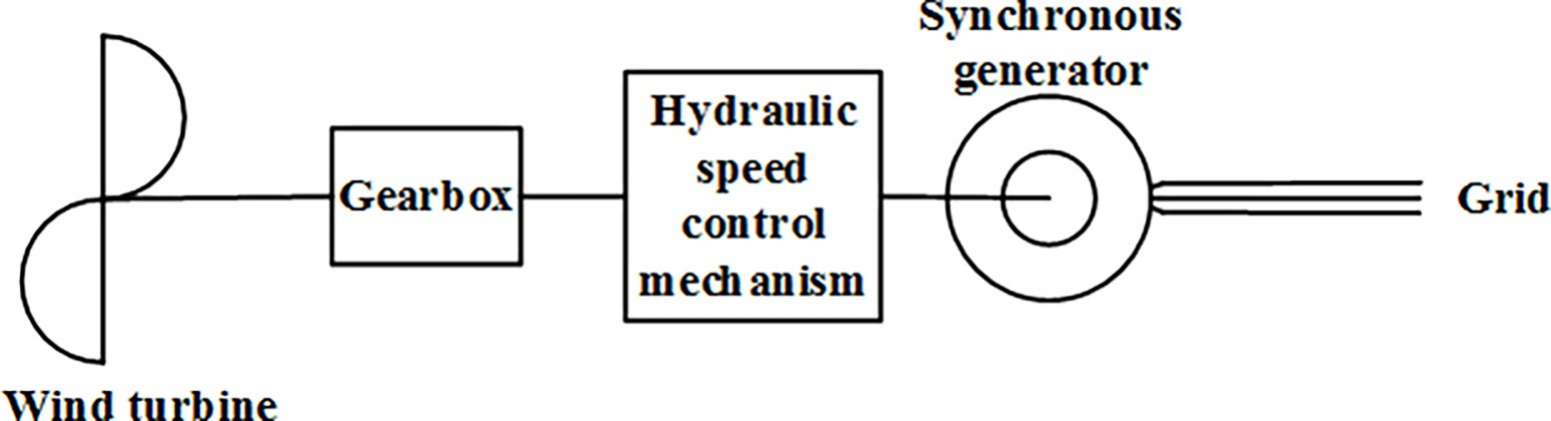
Structural diagram of the hydraulic drive speed regulating wind turbine.

The structural schematic of the hydraulic drive speed regulating wind turbine is shown in Fig 2 [22–24].

The hydraulic drive speed regulating wind turbine offers advantages such as rapid response, high torque, stability, and reliability. However, its complex structure and the hydraulic system’s stringent sealing requirements result in excessive costs, preventing widespread adoption.

### (3) Continuously speed regulating wind turbine

**Fig 3:**
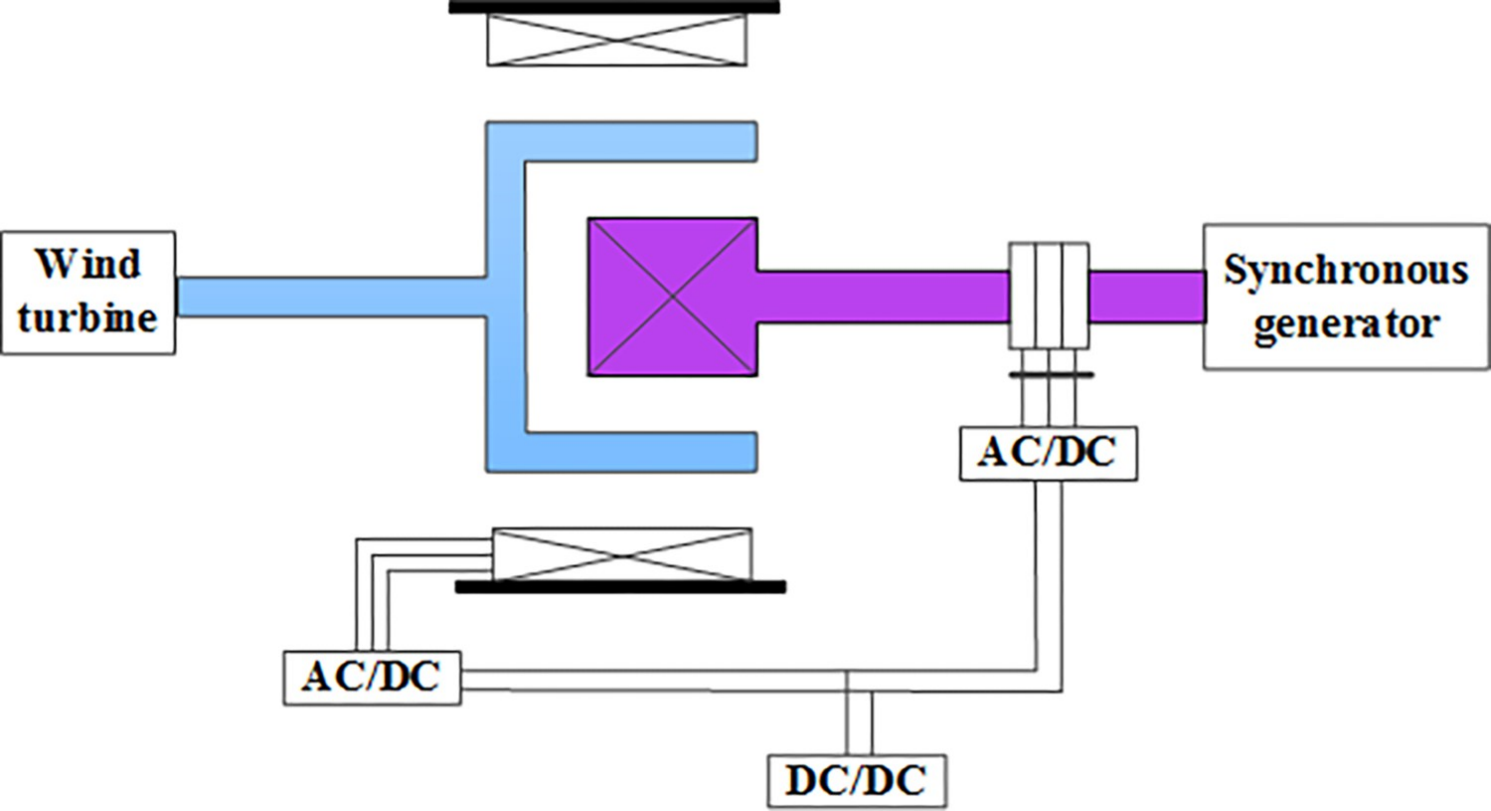
Structural diagram of the continuously speed regulating wind turbine.

Reference [25,26] presents an electrically driven continuously speed regulating wind power generation system, with its structural schematic shown in Fig 3.

Compared to mechanical speed regulating systems, the continuously speed regulating wind turbine exhibits higher reliability. However, the complex structure of the continuously variable transmission and the need for power devices that match the wind turbine’s power result in oversized converter capacities for large wind turbines, thus limiting their application.

### (4) Electromagnetic coupling speed regulating wind turbine

**Fig 4:**
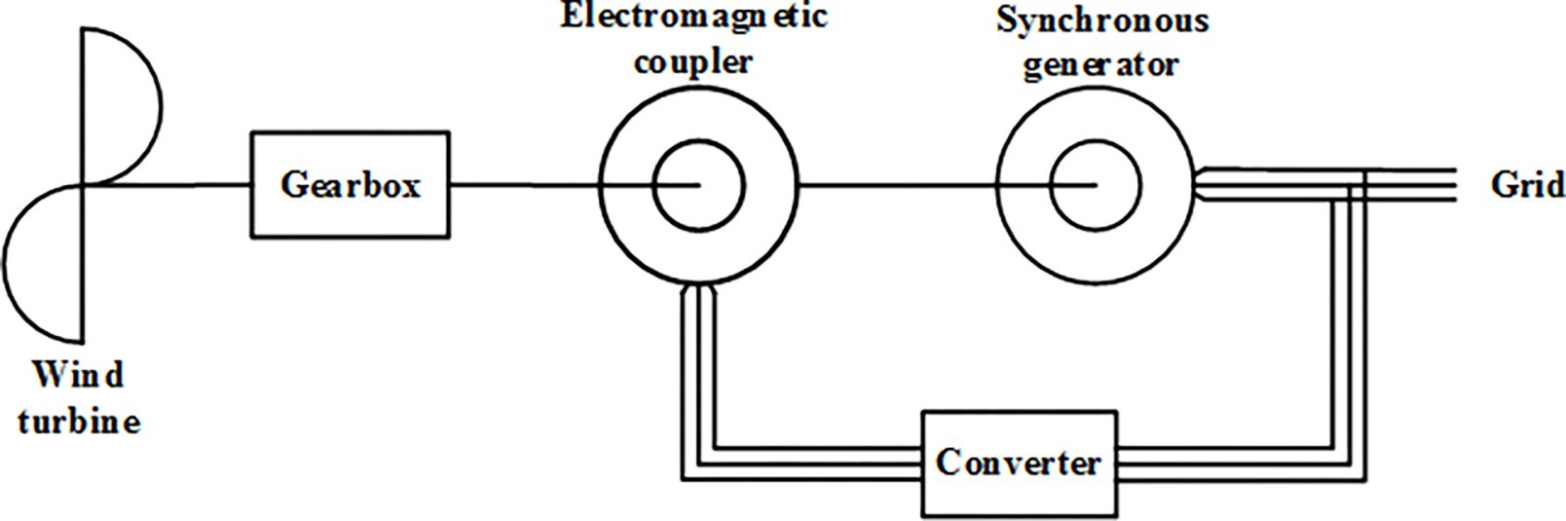
Structural diagram of the electromagnetic coupling speed regulating wind turbine.

**Fig 5:**
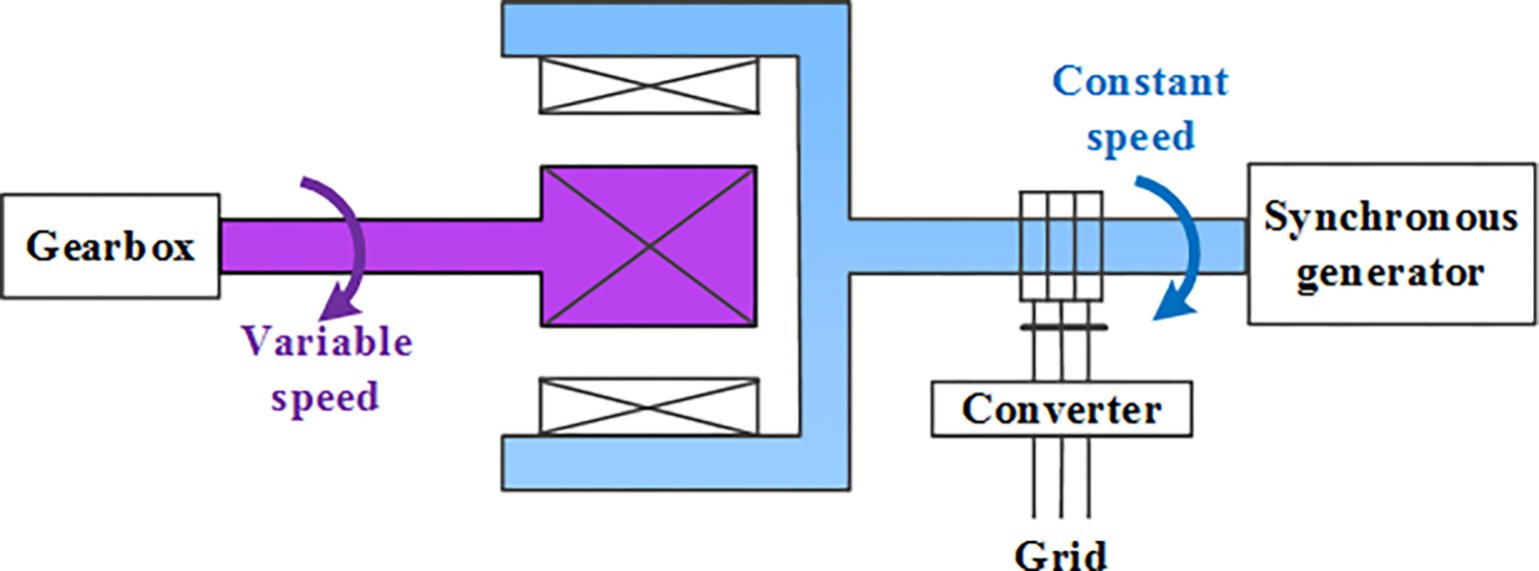
Basic structural schematic of the electromagnetic coupler.

Reference [27–29] developed an electromagnetic coupling speed regulating synchronous wind turbine, with its structural schematic shown in Fig 4. The basic structure of the electromagnetic coupler is illustrated in Fig 5.

The electromagnetic coupler is essentially a wound asynchronous machine; therefore, the electromagnetic coupling variable speed wind turbine functions by utilizing an asynchronous machine with the same capacity as the synchronous generator solely for speed regulation, while the power generation function is performed independently by the synchronous generator. This configuration results in the turbine’s size and weight being twice that of the synchronous generator, complicating transportation and installation, while the power output remains unchanged, indicating that power generation efficiency needs improvement.

Existing variable speed wind turbines achieve flexible connections in the transmission chain through various mechanical and electrical methods, alleviating the impact of shock loads on the turbines to some extent. By incorporating speed regulation devices into the traditional transmission chain, the synchronous generator at the end can connect to the grid without a converter, thus avoiding harmonic pollution caused by power electronic devices and improving the quality of the electrical energy output from wind turbines. Additionally, the synchronous generator at the end of the transmission chain employs mature excitation technology, enabling the variable speed wind turbine to provide sufficient reactive power during grid faults and ensuring low voltage ride-through capability, thus safeguarding the grid’s safe and stable operation.

However, current speed regulating wind turbines still face challenges such as difficulties in component processing, technology promotion issues, oversized converter power, and low power generation efficiency. This research aims to address the problems faced by existing speed regulating wind turbines. Firstly, the proposed wind power system consists of mature machine components, including a permanent magnet outer rotor, a wound inner rotor, and an electrically excited synchronous generator, which can tackle the challenges of component processing and technology promotion. Secondly, in this system, only the stator free speed regulating machine requires a matching power converter, while the synchronous generator does not, classifying it as a partial power converter wind turbine, thus resolving the issue of oversized converter power in existing speed regulating wind turbines. Lastly, the decoupled control strategy for the stator free speed regulating machine that operates at a constant frequency across the full wind speed range addresses the low power generation efficiency of current speed regulating wind turbines.

In the proposed stator free speed regulating wind turbine generation system, the key component, the stator free speed regulating machine, consists solely of two counter-rotating inner and outer rotors without a stator. Due to this structural difference from traditional machines, existing control strategies for conventional wind turbines will not be applicable, necessitating the development of new control strategies suitable for this machine.

The section 2 of this paper presents the structural form and mathematical model of the stator free speed regulating wind turbine generation system. The section 3 investigates the vector control strategy based on the outer rotor magnetic flux orientation for the current inner loop and speed outer loop of the stator free speed regulating machine. And the section 4 validates the feasibility of the stator free speed regulating wind turbine generation system structure and the effectiveness of the control strategy through simulation experiments.

## 2. Proposal of the stator free speed regulating wind turbine generation system

**Fig 6:**
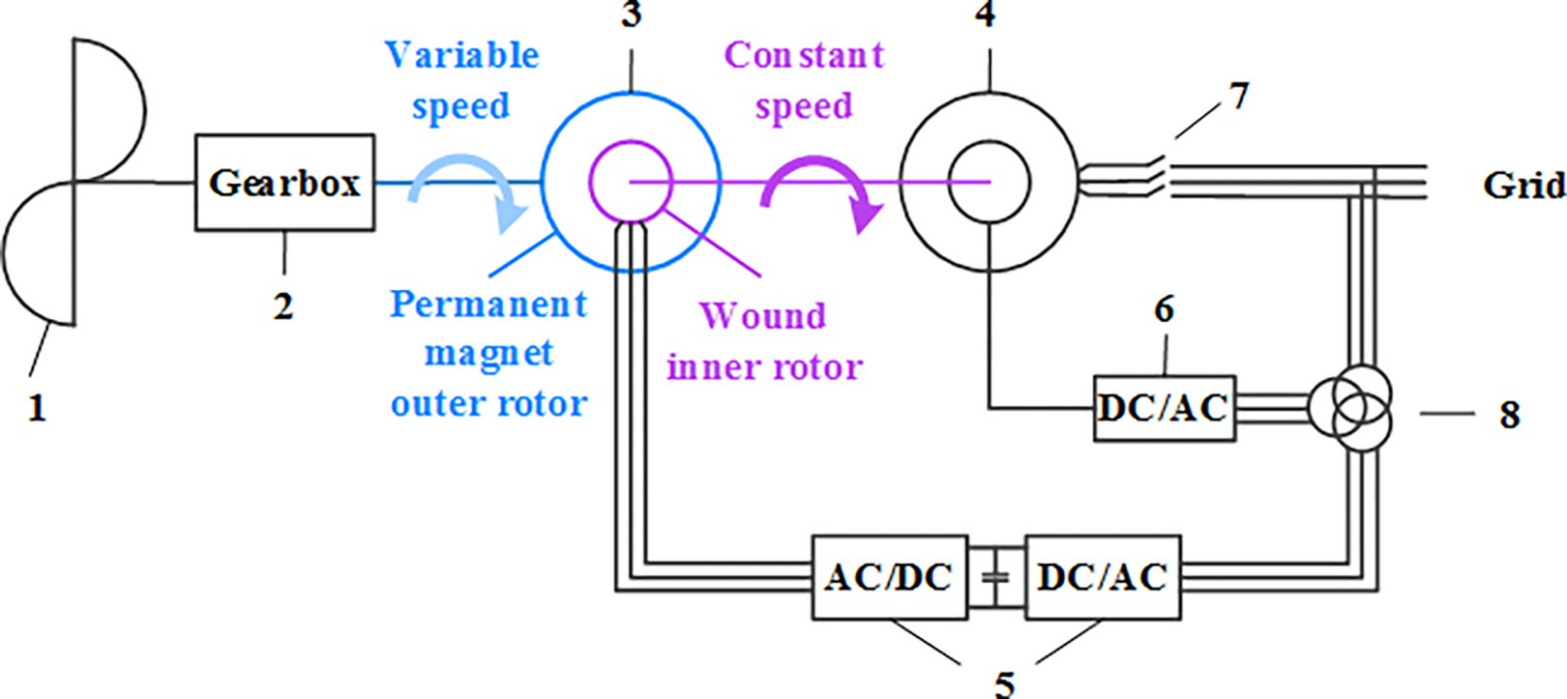
Structure diagram of the stator free speed regulating wind turbine generation system.

The authors have designed a new type of stator free speed regulating wind turbine generation system (Experiment parameters are shown in Supporting file), as shown in the Fig 6. The analysis of the working mechanism of the system and the establishment of the mathematical model are detailed in the authors’ other two papers [30,31]. This article will directly quote the conclusions of the above two papers.

The stator free speed regulating wind turbine generation system is mainly composed of 1—wind wheel, 2—speed increasing gearbox, 3—stator free speed regulating machine (SFSRM), 4—electrically excited synchronous generator (EESG), 5—back-to-back PWM converter, 6—DC-AC converter, 7—main switch and 8—transformer. The stator free speed regulating machine is composed of an outer rotor with a permanent magnet and an inner rotor with the winding. There is no stator in this machine.

The stator free speed regulating wind turbine generation system utilizes a stator free speed regulating machine as the front-end speed adjustment device, which is connected to a gearbox and an electrical excitation synchronous generator at the end of the drive chain. This configuration achieves a flexible connection within the drive chain, enhances the inertia of the wind turbine, mitigates the adverse effects of impact loads on the system to some extent, and improves the frequency stability of the power system. The electrical excitation synchronous generator at the end of the drive chain can directly connect to the grid without the converter, thereby avoiding harmonic pollution caused by power electronic devices and improving the quality of electrical energy output from the wind turbine, which in turn enhances the voltage stability of the power system. Additionally, the mature excitation technology employed in the electrical excitation synchronous generator within the stator free speed regulating wind turbine generation system allows it to provide sufficient reactive power during grid faults, endowing it with low voltage ride-through capability, thus ensuring the safe and stable operation of the power system.

The mathematical model of the stator free speed regulating machine in the two-phase rotating coordinate system is shown in Eq (1) [30].


$$\{\begin{array}{l} u_{d}=Ri_{d}-\omega_{e}L_{q}i_{q}+L_{d}\frac{di_{d}}{dt}\\ u_{q}=Ri_{q}+\omega_{e}L_{d}i_{d}+L_{q}\frac{di_{q}}{dt}+\omega_{e}\lambda_{f} \end{array}$$ (1)

In the equation, *u*_*d*_, *u*_*q*_ and *i*_*d*_, *i*_*q*_ represent the voltage and current of the inner rotor winding along the *d*’, *q*’ axis, respectively. *L*_*d*_, *L*_*q*_ represent the inductance of the inner rotor winding along the *d*’, *q*’ axis, respectively. R represents the resistance of the inner rotor winding. *ω*_*e*_ represents the electrical angular velocity of the rotating magnetic field on the inner rotor, while *λ*_*f*_ represents the magnetic flux of the outer rotor permanent magnet.

The stator free speed regulating machine features independent excitation windings that enable the application of excitation and adjustment of the power factor. The adjustable parameters include the amplitude, frequency, and phase of the excitation current in the inner rotor. By controlling the amplitude of the inner rotor’s excitation current, the reactive power output of the stator free speed regulating machine can be adjusted. Unlike traditional synchronous machines, the stator free speed regulating machine can adjust its speed by controlling the frequency of the inner rotor’s excitation current; by controlling the phase of the excitation current, it can displace the position of the rotating magnetic field generated by the inner rotor current within the air gap space, changing the power angle of the machine and regulating the active power output to achieve generation. Depending on the varying speeds of the inner and outer rotors, the stator free speed regulating machine can operate as both a motor and a generator under different conditions, enabling variable speed constant frequency operation across the entire wind speed range.

The key to controlling the stator free speed regulating machine is to control its electromagnetic torque by controlling the current, and thus achieve the target speed. However, the stator free speed regulating machine is a high-order, nonlinear, strongly coupled multivariable system, with coupling between the inner and outer rotor magnetic fields. It is not possible to directly control the electromagnetic torque by controlling the current. Therefore, a vector control strategy is needed. In the coordinate system of magnetic field orientation, the three-phase current vector of the winding is decomposed into two orthogonal current components through coordinate transformation. One is the excitation current component (d-axis component), which generates the magnetic field, and the other is the torque current component (q-axis component), which generates the torque. Independent control of these two orthogonal current components can achieve decoupling of excitation current and torque current. That is, decoupling of magnetic flux and electromagnetic torque. The key to vector control is to accurately control the electromagnetic torque of the stator free speed regulating machine in steady-state and dynamic conditions by controlling the amplitude and phase of the current.

## 3. Outer rotor flux-oriented vector control strategy for the stator free speed regulating machine

The vector control of the stator free speed regulating machine involves the reconstruction of its mathematical model into that of a separately excited DC motor through vector transformation. This is achieved by transforming the alternating current of the inner rotor into two direct current components: one for excitation (in the direct axis) and the other for torque (in the quadrature axis), which are mutually perpendicular in space. Decoupled control of these two components is then implemented to achieve separate control of the excitation magnetic field and electromagnetic torque of the stator free speed regulating machine in a reference axis system where the two phases rotate relative to each other.

By employing speed and flux control, a torque control loop is established within the speed loop to suppress the influence of flux variation on speed, thereby achieving approximate decoupling of speed and flux and obtaining higher dynamic and static performance. Additionally, decoupling of the current torque component and excitation component is achieved, allowing for the separate design of speed and flux regulators based on linear system theory. Continuous control is implemented, resulting in a wider speed range.

This paper presents an outer rotor flux-oriented vector control strategy for the stator free speed regulating machine. Due to the decoupling of the *d*’ axis and *q*’ axis current components in the stator free speed regulating machine under the *dq*’ rotating coordinate system, aligning the *d*’ axis with the axis of the magnetic linkage of the outer rotor permanent magnet can achieve directed vector control of the outer rotor magnetic field. By adopting *i*_*d*_ = 0 directed vector control of the outer rotor magnetic field, all the currents on the inner rotor are used to generate electromagnetic torque, optimizing the control of electromagnetic torque. As electromagnetic torque has a linear relationship with current and is barely affected by the machine’s inductance parameters, the control of electromagnetic torque has been significantly simplified.

The voltage equation of the stator free speed regulating machine in the *dq*’ coordinate system is as shown in the Eq (2) [30], at steady state:

$$\{\begin{array}{l} u_{d}=Ri_{d}-\omega_{e}L_{q}i_{q}\\ u_{q}=Ri_{q}+\omega_{e}L_{d}i_{d}+\omega_{e}\lambda_{f} \end{array}$$ (2)

When using *i*_*d*_ = 0 control strategy, the voltage equation can be expressed as Eq (3):

$$\{\begin{array}{l} u_{d}=-\omega_{e}L_{q}i_{q}\\ u_{q}=Ri_{q}+\omega_{e}\lambda_{f} \end{array}$$ (3)

**Fig 7:**
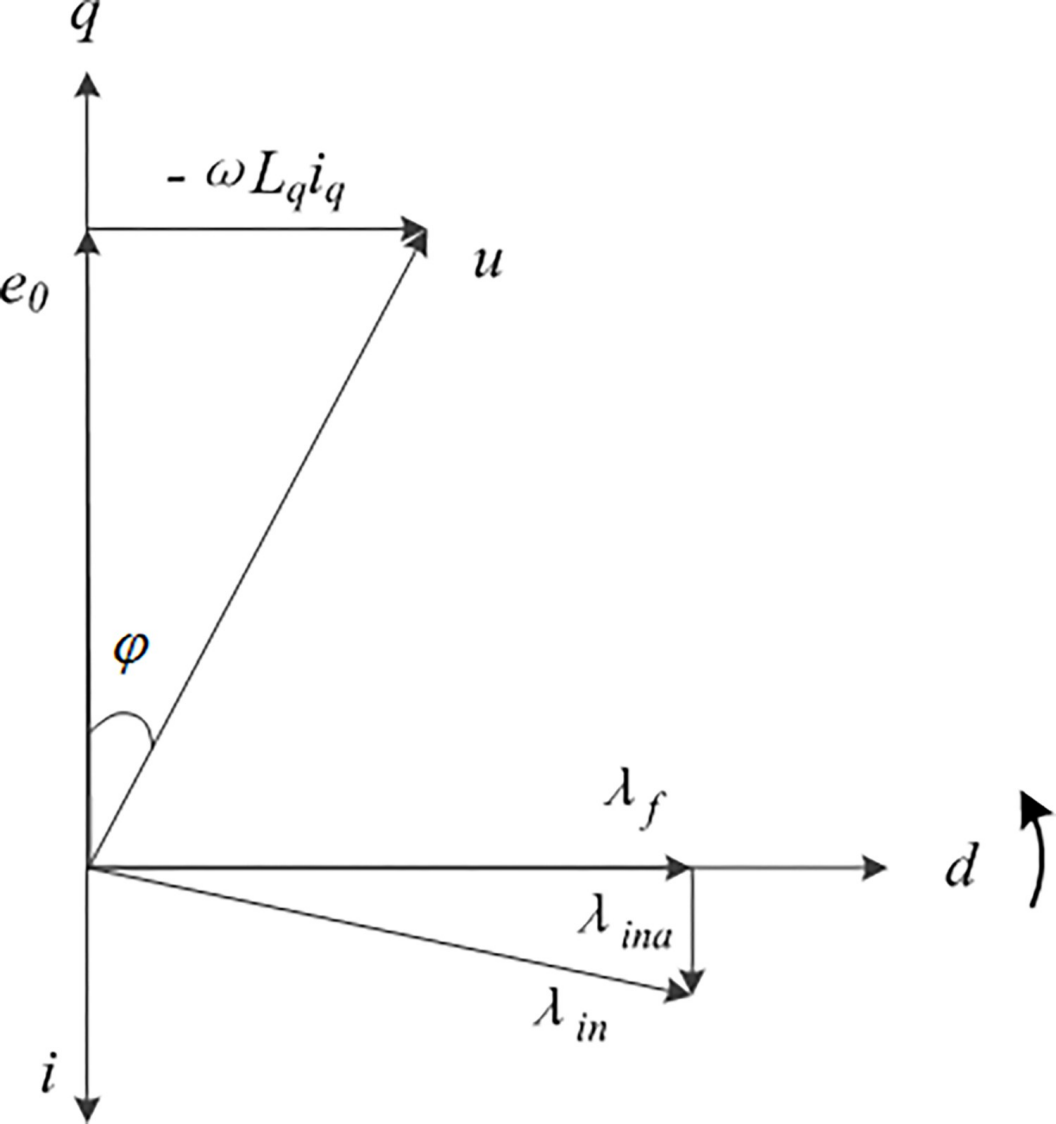
Phase diagram of the stator free speed regulating machine.

The phasor diagram of the stator free speed regulating machine is depicted in Fig 7. The flux resulting from armature reaction is denoted as *λ*_*ina*_, while the flux synthesized by the air gap is denoted as *λ*_*in*_ The non-existence of armature reaction in the direct-axis armature current component, which is shown to be zero, obviates the demagnetizing effect on the permanent magnet, thus constituting a significant advantage. In Fig 7, *φ* represents the power factor angle on the rotor side of the stator free speed regulating machine.

When the torque command *T*_*eref*_ is given, the current command of the *dq*’ axis of the stator free speed regulating machine is as Eq (4) [31]:

$$\{\begin{array}{l} i_{\mathrm{dref}}=0\\ i_{qref}=\frac{2}{3}\frac{T_{\mathrm{eref}}}{p\lambda_{f}} \end{array}$$ (4)

Where, *i*_*dref*_ represents the d axis command current, *i*_*qref*_ represents the q axis command current, p represents the number of pole pairs for the stator free speed regulating machine.

In the motor control, current loop control is a critical component that directly influences the response speed and operational efficiency of the motor. The inductive elements within the motor model introduce control lag, which poses a significant challenge in motor control. The Proportional-Integral (PI) controller effectively addresses this issue. The proportional control component rapidly responds to system deviations, providing a control action that is proportional to the deviation, thereby ensuring that the system can quickly adjust the control variable in the face of disturbances and maintain dynamic balance. Meanwhile, the integral control component gradually eliminates the steady-state error caused by inductance by accumulating the error signals. Even when the deviations are very small, this continuous control action persists until the error is fully eradicated, ensuring the long-term stability and accuracy of the current loop. Furthermore, the design of the PI controller is straightforward, allowing for intuitive parameter adjustments, making it easy to implement and maintain.

**Fig 8:**
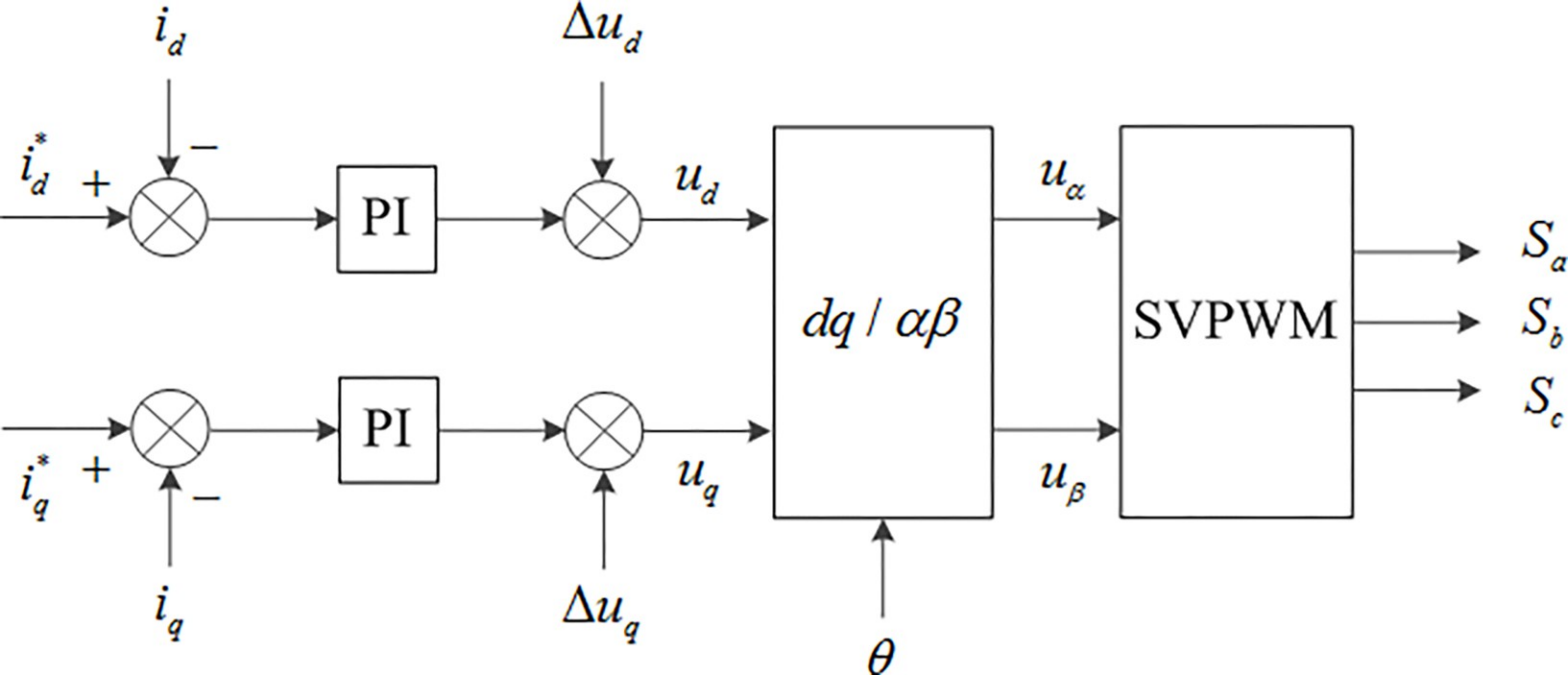
Current control diagram of the stator free speed regulating machine.

Therefore, in the control of the inner rotor current in the stator free speed regulating machine, a PI controller has been introduced to the *dq*’ axis current closed-loop control, in order to acquire a suitable current response. The *dq*’ axis current control equation is depicted in Eq (5) [31], where Δ*u*_*d*_ = −*ωL*_*q*_*i*_*q*_ and Δ*u*_*q*_ = −*ωL*_*d*_*i*_*d*_+*ωλ*_*f*_ act as feedforward compensators, enhancing the dynamic decoupling performance between the system voltage and current. *K*_*P*_, *K*_*I*_ represent the proportional and integral coefficients of the current loop. The overall current control diagram is illustrated in Fig 8.


$$\{\begin{array}{l} u_{d}=\Delta u_{d}+K_{P}(i_{\mathrm{dref}}-i_{d})+K_{I}\int (i_{\mathrm{dref}}-i_{d})dt\\ u_{q}=\Delta u_{q}+K_{P}(i_{\mathrm{qref}}-i_{q})+K_{I}\int (i_{qref}-i_{q})dt \end{array}$$ (5)

From Fig 8, it can be deduced that by comparing the reference value $i_{d}^{*}$ and actual value *i*_*d*_ of the *d* axis current of the inner rotor and applying the feedforward compensation term Δ*u*_*d*_ after PI regulation, the *d* axis voltage value is obtained. Similarly, by comparing the reference value $i_{q}^{*}$ and actual value *i*_*q*_ of the *q* axis current of the inner rotor and applying the feedforward compensation term Δ*u*_*q*_ after PI regulation, the *q* axis voltage value is obtained. Subsequently, the *dq* axis voltage value is converted through *dq*→*αβ* transform to yield the drive signal for the stator free speed regulating machine converter.

As the power, torque and rotating speed of the stator free speed regulating machine satisfy as Eq (6) [31]:

$$P_{e}=T_{e}\omega_{e}$$ (6)

Where, *P*_*e*_ represents the electrical power of the stator free speed regulating machine, *T*_*e*_ represents the electrical torque of the stator free speed regulating machine.

**Fig 9:**
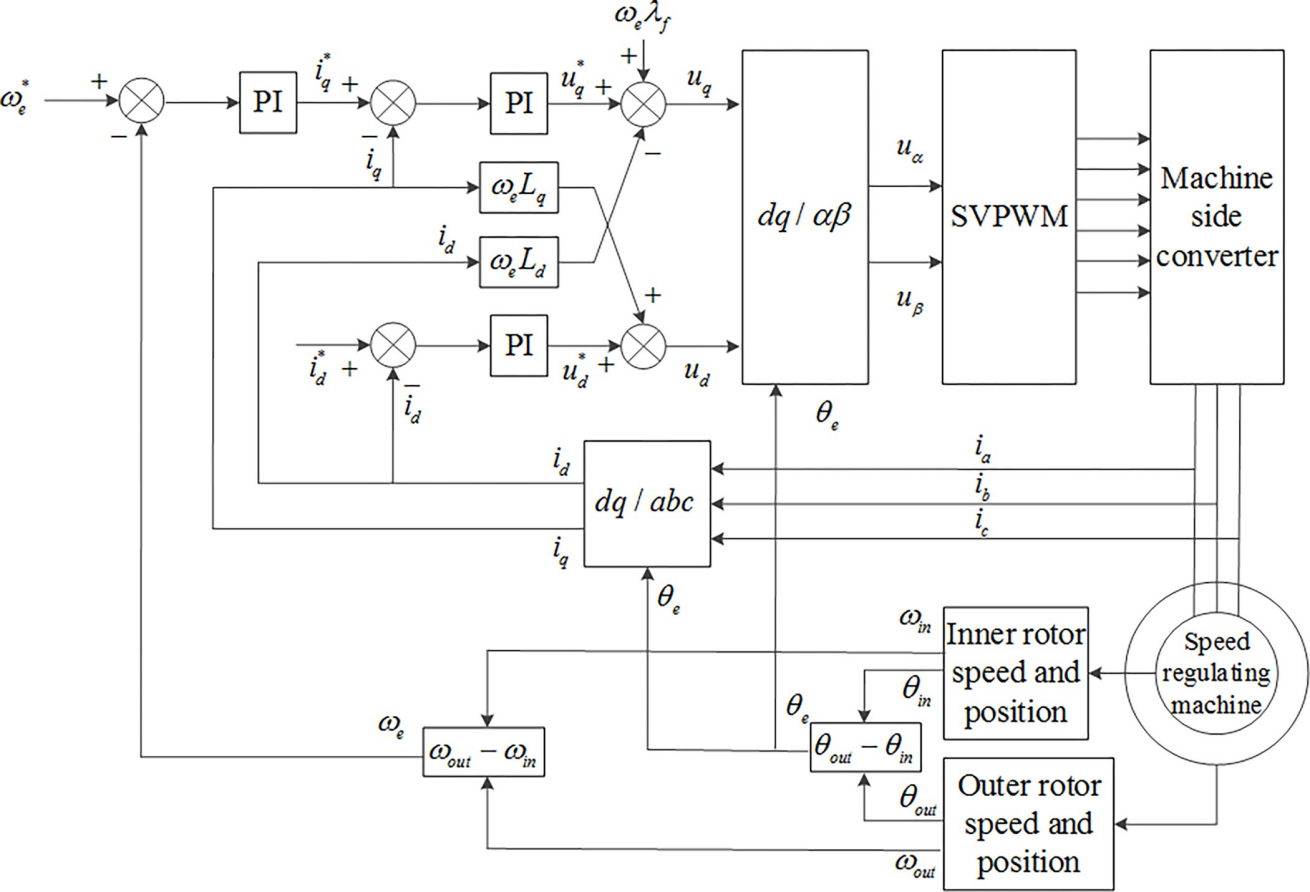
Vector control diagram of the stator free speed regulating machine.

It can be inferred that by regulating the *q*’ axis current component, the electromagnetic torque and power of the stator free speed regulating machine can be controlled. The control diagram of the stator free speed regulating machine is shown in Fig 9. The control principle is based on a control structure with speed control as the outer loop and current control as the inner loop, which ensures maximum power output delivered to the DC bus.

The vector control system of the stator free speed regulating machine, which includes a current loop, enables real-time control of the inner rotor current based on the outer rotor position, dynamically meeting the load requirements for electromagnetic torque. During the start-up of the stator free speed regulating machine, all currents can be used to generate electromagnetic torque, fully utilizing the machine’s overload capacity and improving the start-up speed. Additionally, the control algorithm is executed once per current sampling period, changing the voltage vector output state. The converter switch frequency is constant and can be set relatively high, which is beneficial for modulating a circular magnetic flux trajectory, producing a sinusoidal current waveform, and minimizing torque ripple.

Based on Fig 9, the inner and outer rotor speeds of the stator free speed regulating machine are detected separately, and the difference is compared with the reference value to form the speed outer loop. The speed deviation is adjusted by a PI controller to obtain the *q* axis current reference value, while the *d* axis current reference value is set to 0. The actual values are compared with the reference values to form the current inner loop. The current deviation is adjusted by a PI controller to obtain the *dq* axis voltage reference value. After adding the cross-coupling voltage, the spatial vector control is used to obtain the drive signal for the inner rotor-side converter.

## 4. Simulation of the control strategy for the stator free speed regulating machine

In order to validate the effectiveness of the proposed outer rotor flux-oriented vector control strategy for the stator free speed regulating machine, this paper intends to assess the system’s performance across a range of wind speeds both before and after its connection to the grid. This evaluation will be facilitated through the development of a MATLAB/Simulink-based simulation model simulating the operation of the stator free speed regulating wind turbine generation system.

**Fig 10:**
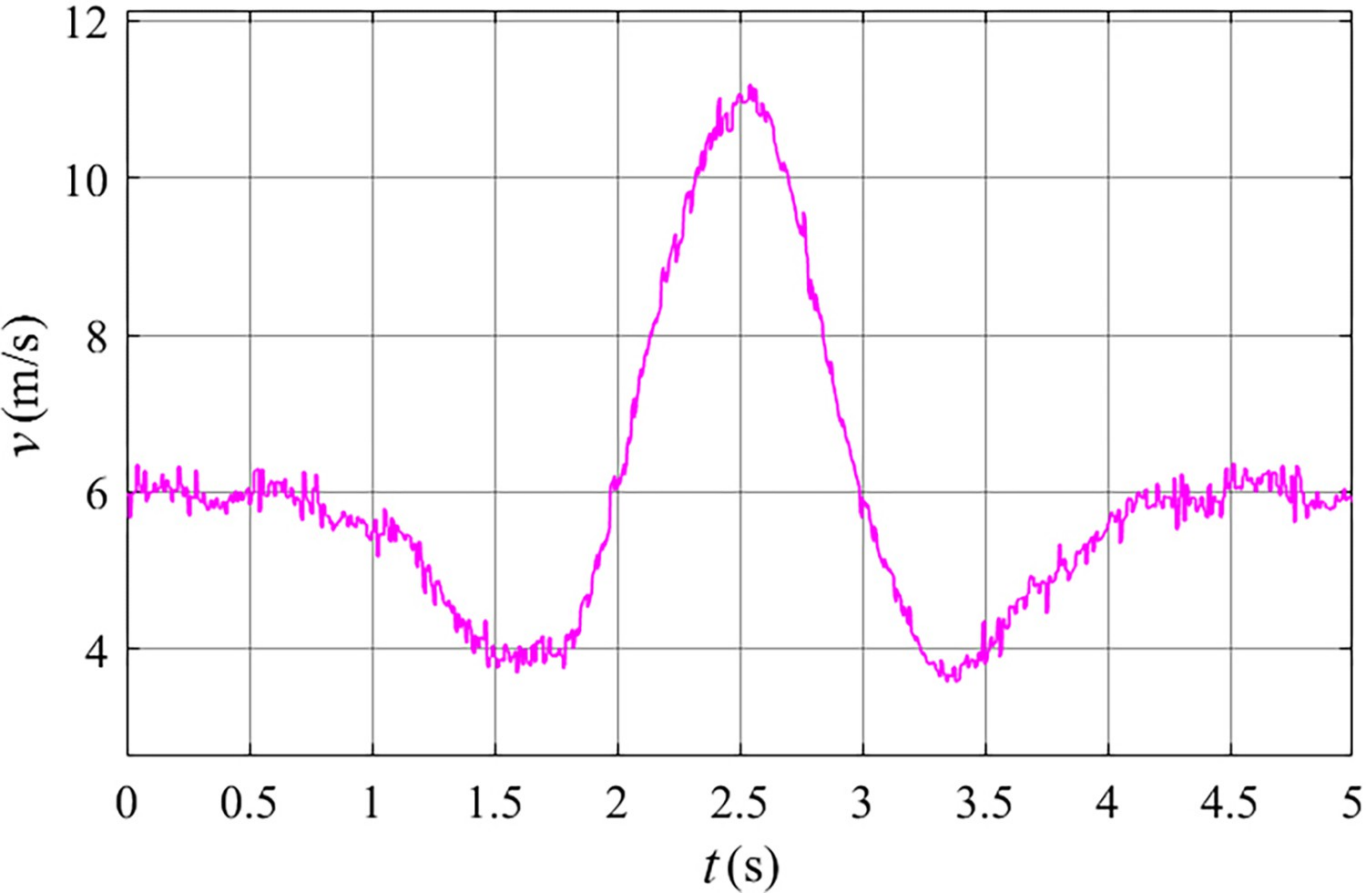
Mexican hat wind speed.

**Fig 11:**
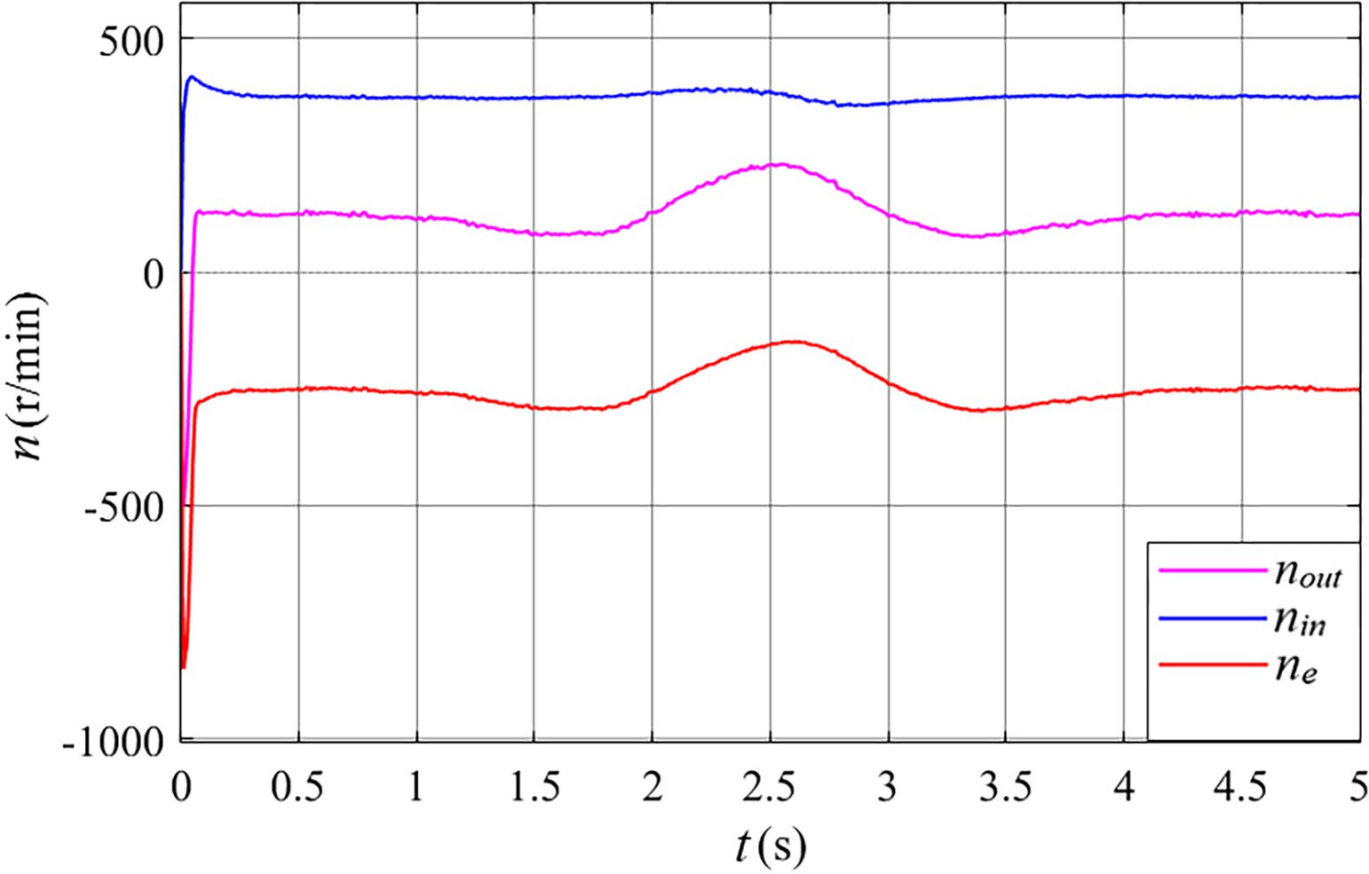
The rotating speed of the stator free speed regulating machine before grid connected in the Mexican hat wind speed.

**Fig 12:**
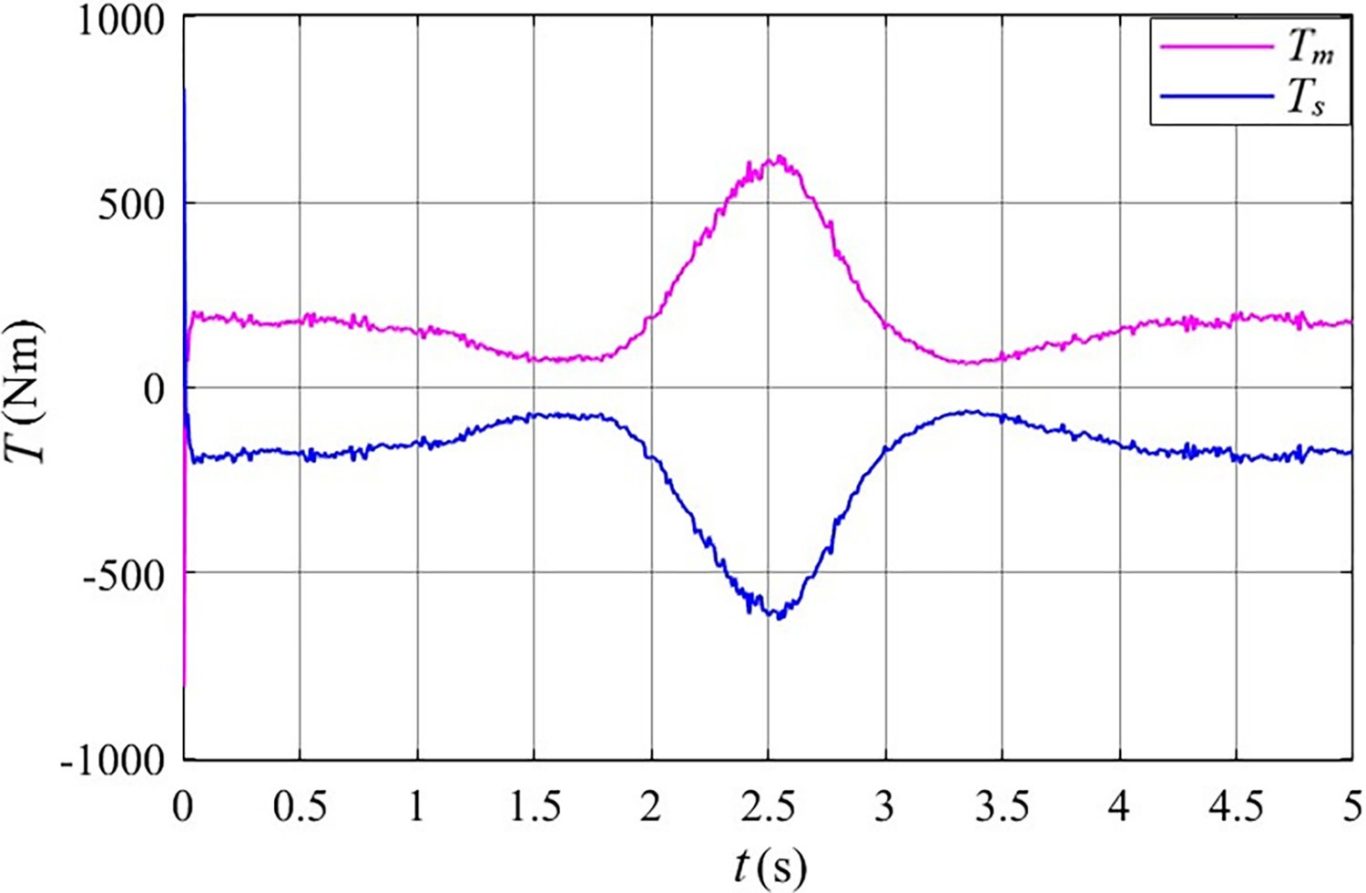
The torque of the stator free speed regulating machine before grid connected in the Mexican hat wind speed.

**Fig 13:**
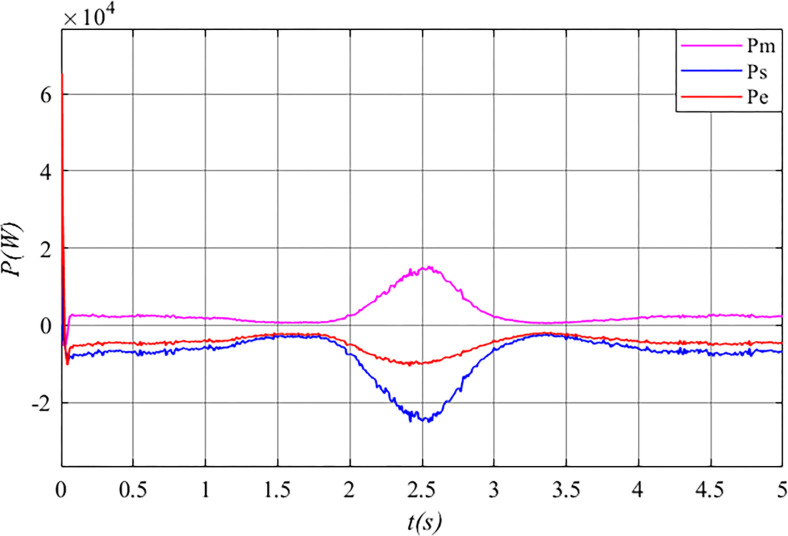
The power of the stator free speed regulating machine before grid connected in the Mexican hat wind speed.

Before meeting the requirements for grid connection, the primary control objective of the stator free speed regulating machine is to achieve synchronous inner rotor velocity. This synchronization is crucial as it enables the Electrically Excited Synchronous Generator (EESG) to directly integrate with the grid without the need for a converter. To validate the proposed strategy, this study has chosen to utilize the Mexican hat wind speed model, illustrated in Fig 10. By employing this model, the relationship between the rotating speed, torque and power of the stator free speed regulating machine is effectively showcased, as depicted in Figs 11 to 13 at this specific wind velocity.

From Fig 11, it can be observed that the inner rotor speed (*n*_*in*_) of the stator free speed regulating machine remains consistently stable at the synchronous speed of 375 r/min. Throughout the entire wind speed range, the outer rotor speed (*n*_*out*_) of the stator free speed regulating machine varies with wind speed, always remaining lower than the inner rotor speed. The rotating magnetic field (*n*_*e*_) of the inner rotor winding has a rotation direction opposite to that of both the inner and outer rotor speeds.

Analysis of Fig 12 indicates that across the full wind speed range, the input mechanical torque (*T*_*m*_) and output mechanical torque (*T*_*s*_) of the stator free speed regulating machine are equal in magnitude but opposite in direction.

Fig 13 illustrates that mechanical power (*P*_*m*_) is input to the stator free speed regulating machine, while mechanical power (*P*_*s*_) is output from it, along with electrical power (*P*_*e*_) being input to the machine. At this point, the stator free speed regulating machine operates in “motor” mode, fulfilling its speed regulation function.

**Fig 14:**
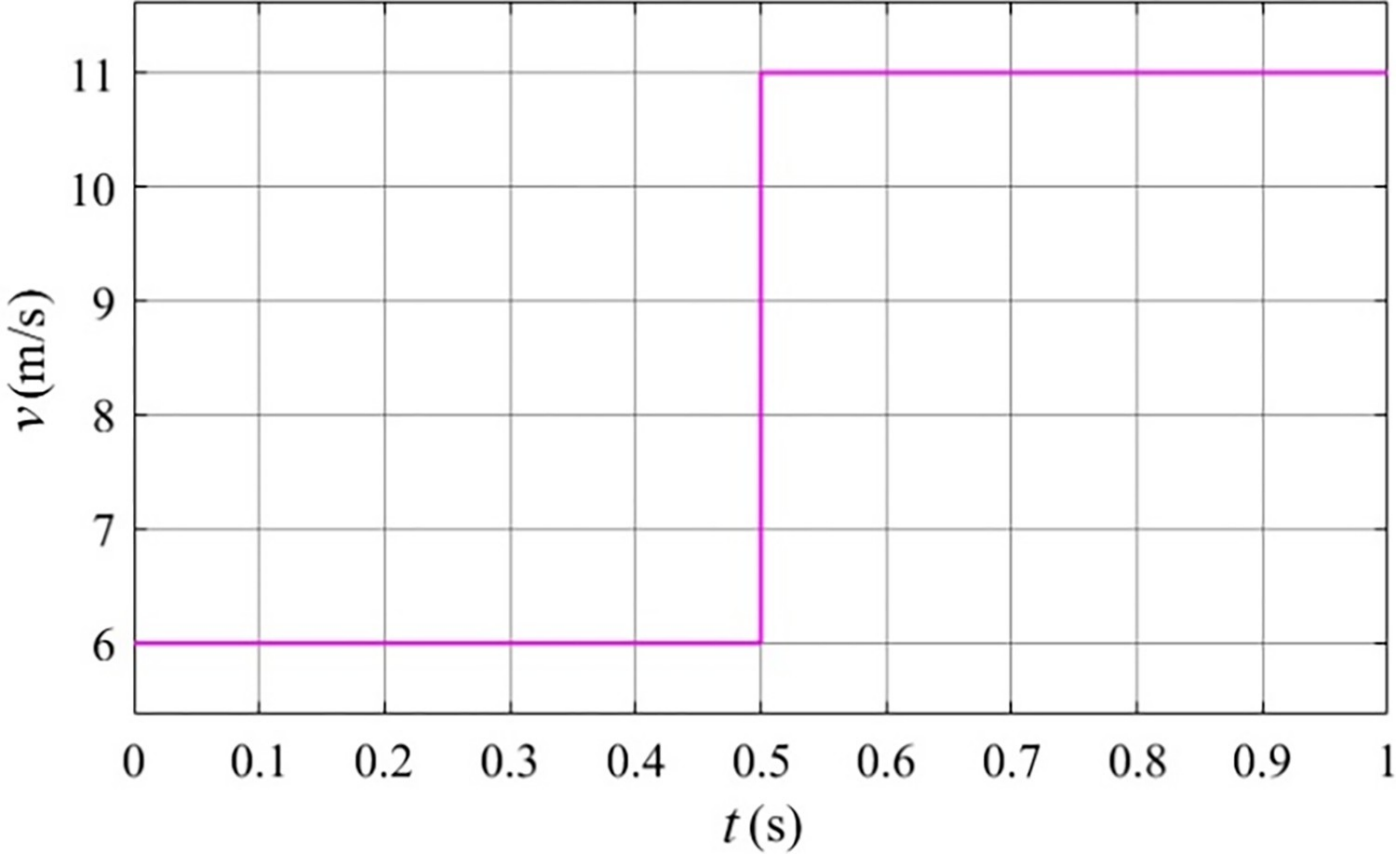
Gust wind speed.

**Fig 15:**
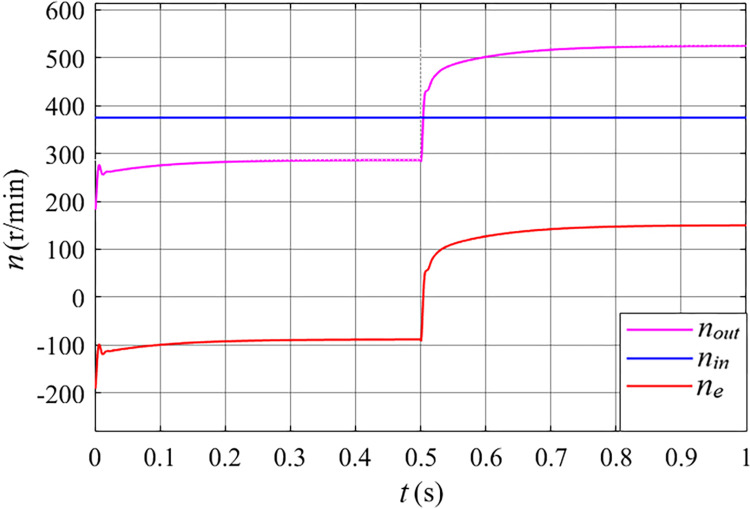
The rotating speed of the stator free speed regulating machine after grid connected in the gust.

**Fig 16:**
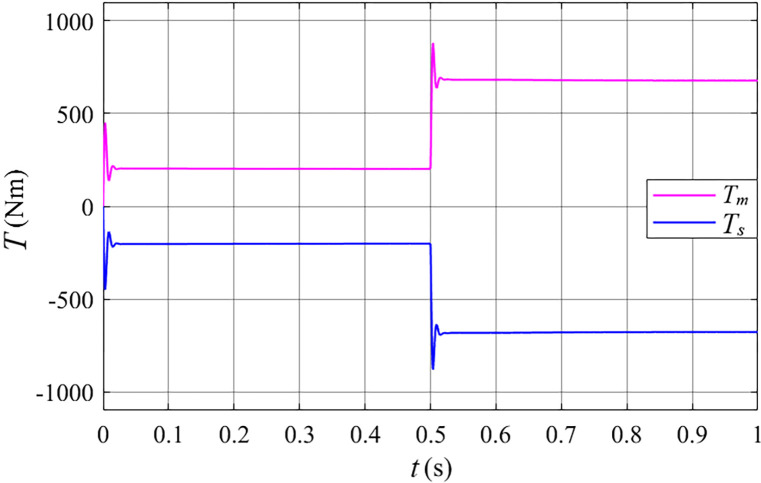
The torque of the stator free speed regulating machine after grid connected in the gust.

**Fig 17:**
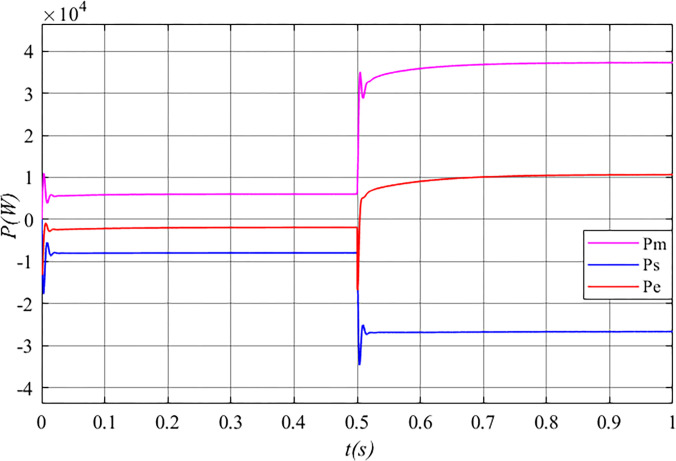
The power of the stator free speed regulating machine after grid connected in the gust.

The primary goal of this investigation is to seamlessly integrate a stator free speed regulating wind turbine generation system with a large grid while ensuring the inner rotor speed remains synchronous. The central objective of the control system is to precisely follow and optimize the outer rotor speed in response to varying wind speeds. To validate the effectiveness of the system, a wind speed model, depicted in Fig 14, is employed for analysis. This model introduces a scenario where the wind speed undergoes an abrupt change from 6 m/s to 11 m/s at the 0.5 second. The rotating speed, torque and power of the stator free speed regulating machine at this specific wind speed are illustrated in Figs 15 to 17. The study aims not only to comprehend but also to harness the maximum wind energy through the implemented control system. Furthermore, the performance of the control system is rigorously examined and validated using the dynamic wind speed model, adding a layer of robustness to the overall study.

From the analysis of Fig 15, it can be concluded that the outer rotor speed (*n*_*out*_) of the stator free speed regulating machine is capable of tracking its optimal speed of 286 r/min when the wind speed is 6 m/s. Following a wind speed change at 0.5 s, the outer rotor speed stabilizes at an optimal speed of 525 r/min within 0.4 s at a wind speed of 11 m/s. Prior to 0.5 s, the outer rotor speed of the stator free speed regulating machine is lower than that of the inner rotor (*n*_*in*_), with the rotating magnetic field (*n*_*e*_) of the inner rotor winding oriented in the opposite direction to the speeds of both the inner and outer rotors. After 0.5 s, the outer rotor speed exceeds that of the inner rotor, and the rotating magnetic field of the inner rotor winding aligns with the direction of both rotor speeds.

Analysis of Fig 16 reveals that across the entire wind speed range, the input mechanical torque (*T*_*m*_) and output mechanical torque (*T*_*s*_) of the stator free speed regulating machine are equal in magnitude but opposite in direction.

In Fig 17, before 0.5 s, mechanical power (*P*_*m*_) is input to the stator free speed regulating machine, while mechanical power (*P*_*s*_) is output from the machine, along with electrical power (*P*_*e*_) being input to it. At this point, the stator free speed regulating machine operates in “motor” mode, performing speed regulation. After 0.5 s, mechanical power (*P*_*m*_) continues to be input, mechanical power (*P*_*s*_) is still output, but electrical power (*P*_*e*_) is now output from the machine. Consequently, the stator free speed regulating machine operates in “generator” mode, fulfilling a power generation role.

## 5. Conclusion

This paper addresses the stability issues of frequency and voltage in high-penetration renewable energy power systems by proposing a novel stator free speed regulating wind turbine generation system. This system comprises a stator free speed regulating machine connected to a gearbox and a synchronous generator, forming a flexible transmission chain that enhances system inertia. Furthermore, the synchronous generator at the end of the transmission chain can connect directly to the grid without the need for a converter, following the speed adjustment by the stator free speed regulating machine. This configuration mitigates harmonic pollution caused by power electronic devices in traditional wind turbines and enhances the reactive power support capability of the system. The increased inertia of the wind turbine generation system and improved reactive power support capacity ensure the frequency and voltage stability of high-penetration renewable energy power systems.

Unlike traditional wind turbines, the proposed stator free speed regulating machine consists solely of a permanent magnet outer rotor and a wound inner rotor, thereby eliminating the stator. Additionally, under varying operating conditions, the stator free speed regulating machine can function as both a motor and a generator, effectively achieving speed regulation and power generation. This unique machine structure renders conventional control strategies ineffective; therefore, this paper presents a control strategy tailored for this machine. By aligning the d-axis in the rotating reference frame with the vector of the magnetic flux axis of the outer rotor’s permanent magnets, vector control of the outer rotor’s magnetic flux can be achieved, employing a dual closed-loop PI control strategy with an outer speed loop and an inner current loop.

To validate the feasibility of the proposed stator free speed regulating wind turbine generation system structure and the effectiveness of the control strategy, this study conducted simulation experiments to analyse the operational states of the stator free speed regulating machine under varying wind conditions, including gusty wind and Mexican Hat wind. The simulation results indicate that prior to grid connection, the stator free speed regulating machine consistently operates in motor mode to achieve speed regulation. After grid connection, at low wind speeds, the machine continues to operate in motor mode; conversely, at high wind speeds, it transitions to generator mode to facilitate power generation. Furthermore, across a full range of wind speeds under various wind conditions, the stator free speed regulating machine successfully maintains variable speed constant frequency operation, thereby enhancing the utilization of wind energy resources.

Future work will focus on further optimizing the control strategy through intelligent control methods and exploring the reactive power support capability of the stator free speed regulating wind turbine generation system, contributing to the stability and reliability of high-penetration renewable energy power systems.

## Supporting information

S1 File Experiment parameters. (DOCX)
