# Supplementary material for: Control strategy of the novel stator free speed regulating wind turbine generation system
Source: PLoS One. 2024 Dec 6;19(12):e0314226. doi: 10.1371/journal.pone.0314226 (PMC11623470; doi:10.1371/journal.pone.0314226)
Supplement: S1 File — (DOCX) [file pone.0314226.s001.docx]

Parameters of the Machine.

| **Parameters** |  | **Value** |
| --- | --- | --- |
| Rated speed $n_{N}$ |  | 575 r/min |
| Rated power $P_{N}$ |  | 48kW |
| Synchronous speed $n_{0}$ | | 375 r/min |
| Inner rotor resistance $R_{in}$ | | 0.013 Ω |
| Inner rotor inductance $L_{d}=L_{q}$ | | 0.0002 H |
| Permanent magnet flux of outer rotor $\lambda_{f}$ | | 0.03 Wb |
| Inertia of outer rotor $J_{out}$ | | 0.1 kgm^2^ |
| Inner rotor inertia  | | 0.1 kgm^2^ |
| Pole pairs$p$ | | 8 |
| **Wind Turbine** | | |
| Synchronous wind speed  | | 8.3 m/s |
| Optimal tip speed ratio  | | 9 |
| Impeller radius$R$ | | 5.6 m |
| Wind Turbine Airfoils  | | 0.48 |
